# Supplementary material for: Morphogenesis of starfish polymersomes
Source: Nat Commun. 2023 Jun 17;14:3612. doi: 10.1038/s41467-023-39305-8 (PMC10276845; doi:10.1038/s41467-023-39305-8)
Supplement: Supplementary file 1 — Supplementary Information [file 41467_2023_39305_MOESM1_ESM.pdf]

Supplementary Information

Morphogenesis of Starfish Polymersomes

Jiawei Sun, Sandra Kleuskens, Jiabin Luan, Danni Wang, Shaohua Zhang, Wei Li, Gizem Uysal, Daniela A. Wilson*

Institute for Molecules and Materials Radboud University Heyendaalseweg 135, 6525 AJ, Nijmegen, the Netherlands

d.wilson@science.ru.nl

Supplementary Methods

Supplementary Method 1. Materials

All reagents and chemicals were purchased from commercial sources and used as received. Styrene (Sigma-Aldrich) was distilled to remove the inhibitor before polymerization. CuBr (Sigma-Aldrich) for ATRP was stirred with glacial acetic acid, then washed with ethanol and diethyl ether under the protection of Argon. Tetrahydrofuran (THF) used in the reaction was distilled from sodium/benzophenone under Argon. MilliQ water obtained from MilliQ QPOD purification system (18.2 MΩ) was used in all the experiments. α-methoxy-ω-hydroxypoly(ethylene glycol) (M_n 2000 g/mol) for synthesis, magnesium sulfate (99%), sodium chloride (99%), ethylenediaminetetraacetic acid (EDTA, 99,4%), 1-phenyl-1-trimethylsiloxyethene, α-bromoisobutyryl bromide (98%), chloroform-*d* ($CDCl_3$, 99,8%), tert-butyl α-bromoisobutyrate and *N,N,N',N'',N''*-Pentamethyldiethylenetriamine (PMDETA, 98%) were purchased from Sigma Aldrich. THF and anisole (99%) were obtained from Acros. MeOH (99,8%), triethylamine (99,5%) and hydrogen peroxide (H_2O_2) was purchased from J.T. Baker. Diethyl ether (99%, Carlo erba Reagents), 1,4- dioxane (Biosolve BV), dichloromethane (CH_2Cl_2, 99,8%) from Thermo Fisher Scientific were also used. THF and Dioxane used for DLS were inhibitor free for HPLC (≥99.9%) purchased from Sigma-Aldrich, $CuSO_4 \cdot 5H_2O$ and Poly(N-isopropylacrylamide)-carboxylic acid terminated with average M_n 10,000 were also purchased from Sigma-Aldrich. Cyanine3 (Cy3, 97%) amine was purchased from Lumiprobe GmbH, and Black Hole Quencher 2 (BHQ2) amine was purchased from Immunosource. Nuclear Magnetic resonance (NMR) characterization was carried out on a Bruker AVANCE HD nanobay console with a 9.4 T Ascend magnet (400 MHz) and a Bruker AVANCE III console with a 11.7 T UltraShield Plus magnet (500 MHz) equipped with a Bruker Prodigy cryoprobe, in $CDCl_3$ or D_2O. NMR spectra were recorded at 298 K unless otherwise specified. Fluorescence spectra were recorded by JASCO FP-8300ST Spectrofluorometer, using excitation at 512 nm and emission from 550-700 nm, with following settings have been used for all measurements: increment = 1 nm, integration time = 0.2 s, bandpass(emission) =

bandpass(excitation) = 2.5 nm. Transmission electron microscopy (TEM) samples were prepared in the following way: a solution of sample (5 µL) was air-dried on a carbon-coated Cu or Ni TEM grid (200 mesh). JEOL TEM 1400 microscope at an acceleration voltage of 120 kV and JEOL TEM 2100 at an acceleration voltage of 200 kV were used for the characterization of shape transformation. (Cryo-)SEM was performed on a JEOL 6,330 Cryo Field Emission Scanning Electron Microscope at an acceleration voltage of 3 kV in cryo-mode and 10 kV in dry mode.

Supplementary Method 2. Experimental Procedures:

Supplementary Method 2.1. Synthesis of poly(ethylene glycol)-polystyrene block copolymer: PEG-*b*-PS_n was synthesized based on our previous work. Briefly, Poly(ethylene glycol) methyl ether (5.00 g, 2.50 mmol) was dried by co-evaporation with toluene and then dissolved in freshly distilled THF in a flamed-dried Schlenk flask. After adding triethylamine (1.04 mL, 7.50 mmol), the solution was cooled to 0 °C, and α-bromoisobutyryl bromide (616 mL, 5.00 mmol) was added dropwise. The solution was then stirred for 24 h while slowly warming to room temperature. The white precipitate was filtered and the solution was concentrated. The polymer was then precipitated in ice cold diethyl ether (3x) and detected by ^{1}H-NMR in $CDCl_3$. After the initiator was synthesized, CuBr (45 mg, 0.32 mmol) was added to the Schlenk tube and kept under vacuum for 15 minutes. After refilling with Argon, PMDETA (66 mL, 0.32 mmol) in anisole (0.5 mL) was added and stirred vigorously for 15 minutes. Styrene (5 mL, 43.6 mmol) in anisole (0.5 mL) was added via a syringe. The solution was cooled to 0 °C and PEG-initiator (215 mg, 0.1 mmol) was added followed with the reaction tube transferred into an oil bath at 90 °C. ^{1}H-NMR was used for monitoring the reaction process. After reaching the desired molecular weight, 1-phenyl-1- trimethylsiloxyethene (1.91 mL, 9.28 mmol) was added to quench the polymerization. The solution was diluted with CH_2Cl_2 and extracted with an aqueous EDTA solution (65 mM). The organic layer was collected, dried with $MgSO_4$ and concentrated. The polymer was obtained after precipitation in MeOH (3x), dried under vacuum overnight and characterized by ^{1}H-NMR in $CDCl_3$ and GPC. The obtained

amphiphilic polymer PEG_{44}-b-PS_{172} has a number average molecular weight (M_w) of 19913.3 g/mol and a PDI of 1.04.

Supplementary Method 2.2 Synthesis of polystyrene-Cy3 and PNIPAm-BHQ2. HOOC-PS 4 (61 mg, 3 μmol) / HOOC-PNIPAm (30 mg, 3 μmol) and Cy3 NH_2 (2 mg, 3 μmol) / BHQ2 NH_2 (2 mg, 3 μmol) were dissolved in DMF (2 mL), and then DiPEA (2 μL, 11.3 μmol) was added. The solution was then stirred at 0 °C for 10 min followed with PyBOP (4.5 mg, 8.5 μmol) added. The reaction was slowly warmed to room temperature while stirring for 4 days, and the reaction was followed by TLC. Sample was then vacuumed overnight to remove DMF and dissolved in Ethyl acetate, centrifuged to remove the undissolved salt, and separate the products with silica columns. Ethyl acetate was the eluent, and the product came out first. PNIPAm-BHQ2 was separated with acetone.

Supplementary Method 2.3 PNIPAm insertion determined by fluorescence spectroscopy. Firstly, polymersomes embedded with PS-Cy3 were prepared by dissolving 9 mg PEG-PS and 1 mg PS-Cy3 in THF : dioxane (4:1 v/v), and followed by water addition and dialysis as described above. After polymersome-Cy3 was formed, 490 μL rigid polymersome solution was transferred to a 5 mL vial, and then 250 μg (5 μL) of PNIPAm-BHQ2 and 30 μL $NaNO_3$ (1 M) were added to the polymersome solution. After mixing for ten minutes, THF : dioxane (4:1 v/v) mixture was added through a syringe pump at the rate of 300 μL h^{-1} at a stirring speed of 900 rpm. The samples were quenched at 30 min, 45 min or 1 hour, and washed by centrifugation 5 times at 10000 x g. The polymersome pellets were then dissolved in water and the fluorescence was measured by Fluorescence Spectroscopy.

Supplementary Methods 2.4 Negative staining of PNIPAm in polymersomes. In order to avoid the influence of free PNIPAm in the solution, particles were separated by centrifugation for 5 times, the upper solution with free PNIPAm was tossed. Then 3 μL of the polymersomes solution was added on top of the carbon-coated 400-mesh copper/Ni grid and absorb for 5 min. The excess fluid was then blotted away with filter paper. 3 μL of $CuSO_4$ (250 mM) solution was added to the top of the grid for staining. After staining for 5 min, the excess fluid was then blotted away with filter paper. Samples were then dried for 1 day before examination.

Supplementary Method 2.5 Isothermal titration calorimetric (ITC) measurement. A MicroCal™ isothermal titration calorimetric (MicroCal PEAQ-ITC Automated, Malvern Panalytical) was used for all experiments. MicroCal PEAQ-ITC analysis software was used for data processing and analysis. The dilution enthalpies for the addition of PNIPAm, PEG or salt to water was used as control groups and removed during data. Samples were prepared in MilliQ water, the sample cell volume was 370 μL. The sample cell was filled with an aqueous solution of polymersomes (0.25 mM/0.5 mM) or polymer. Each titration step was defined by the injection volume (typically 5 μL), syringe solution concentration was adjusted for each experiment.

Supplementary Method 2.6 Energy Dispersive X-ray (EDX) Spectroscopy Measurements. Energy dispersive X-ray spectroscopy was used for the chemical elements on the surfaces of all implant samples. The carbon (C), oxygen (O), copper (Cu), Nitrogen were detected.

Supplementary Method 2.7 Parameterization of shapes and calculations. The parameterization in this manuscript is done according to our previous work[1], in which the reduced volume, v is defined as :

(1) $\boldsymbol{v} = \mathbf{V}/(\mathbf{4}/\mathbf{3}\boldsymbol{\pi}(\frac{\mathbf{A}}{\mathbf{4}\boldsymbol{\pi}})^{\mathbf{3}/\mathbf{2}})$

With A the surface area, of a closed parameterized shape, V the volume of a closed parameterized shape.

The area difference of a vesicle is defined as:

(2) $\Delta\mathbf{A} = \mathbf{4}\boldsymbol{d}\oint \boldsymbol{H}\boldsymbol{d}\mathbf{A}$

With H the mean curvature which can be written in terms of the parameterization, $\boldsymbol{d}$ is the distance between the midplanes of the whole membrane and the midplane of one monolayer, which corresponds to ¼th of the membrane thickness.

The reduced area difference, $\Delta\boldsymbol{a}$, is then defined as:

(3) $\Delta\boldsymbol{a} = \Delta\mathbf{A}/(\mathbf{16}\boldsymbol{\pi}\boldsymbol{d}\sqrt{\frac{\mathbf{A}}{\mathbf{4}\boldsymbol{\pi}}})$

Supplementary Figures

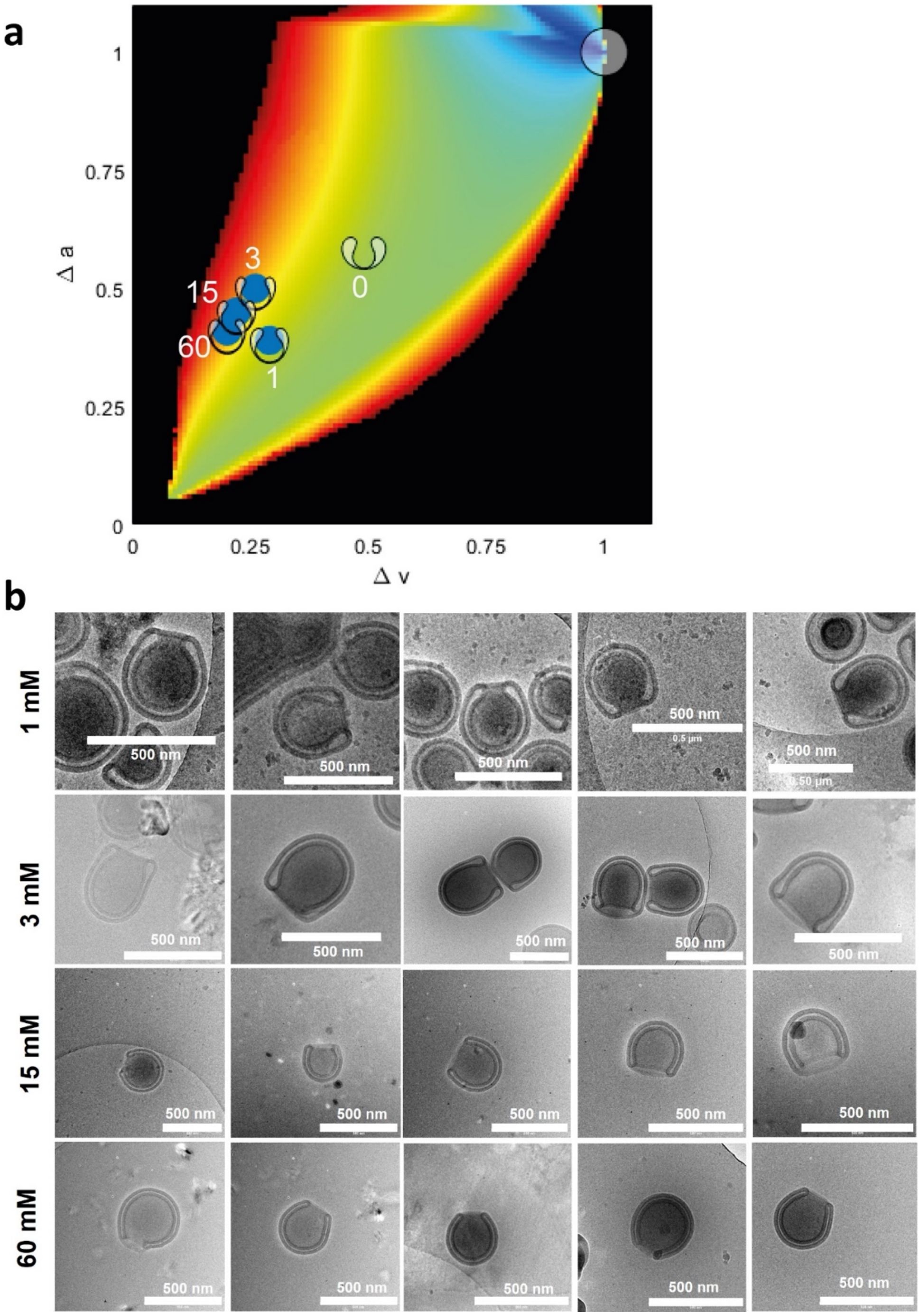

Supplementary Figure 1. (a) Phase diagram showing the positions of the stomatocytes with different amount of salt (0-60 mM) added in the shape transformation using the reduced volume (Δv) and the reduced area difference ($\Delta\boldsymbol{a}$) . For every fitted shape, 5 structures were used, the reduced area difference and the reduced volume are calculated and averaged. The colour scale in the phase diagram indicates the minimized bending energy (Ebend/k) for each reduced volume–reduced area difference combination. (b) Cryo-TEM images of the 5 structures used for parametrization from each salt concentration. Scale bar 500 nm.

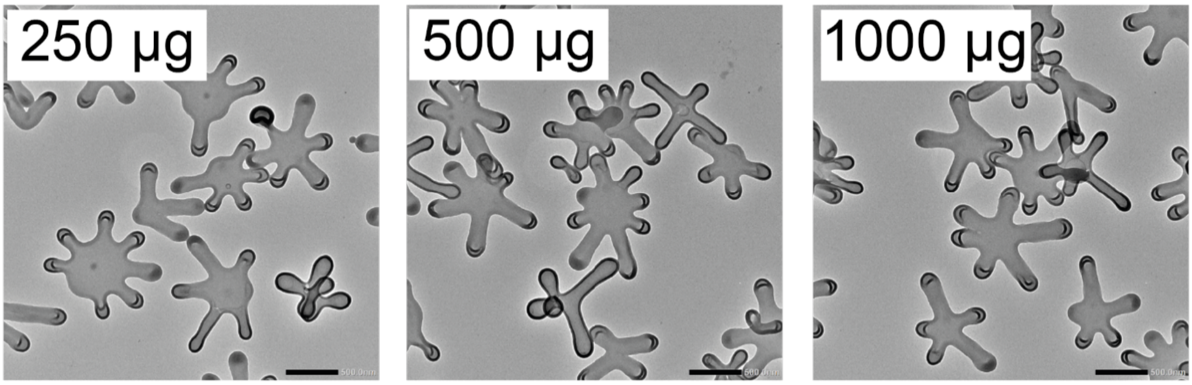

Supplementary Figure 2. Shape transformation of polymersomes with the amount of PNIPAm varies. 250 µg, 500 µg, 1000 µg of PNIPAm were added to different samples together with 15 mM $NaNO_3$. When 23% of organic solvent were added into the systems, samples were quenched and examined by TEM, polymersomes has changed to starfish-like shapes with mostly 5 arms. The increase of PNIPAm didn't make a difference in the shape transformation, as PNIPAm is saturated in the solution. Scale bar 500 nm.

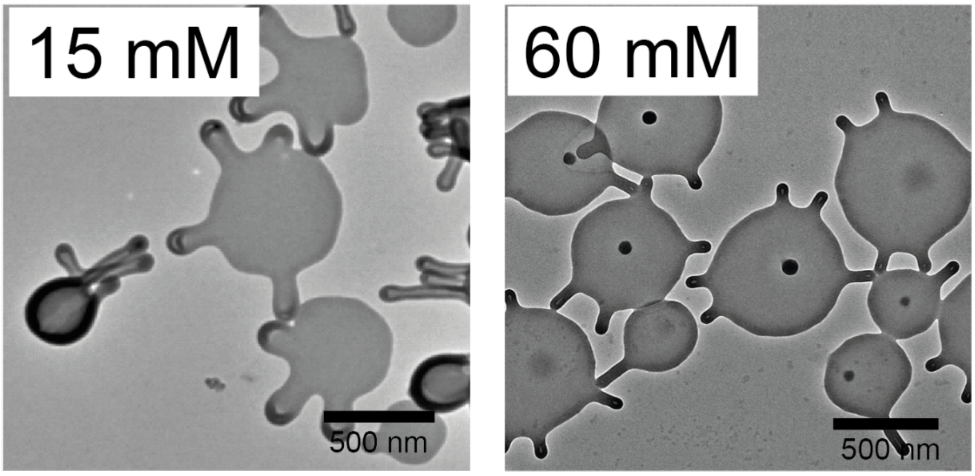

Supplementary Figure 3. Shape transformation of polymersomes with the amount of salt varies. 15 mM or 60 mM of $NaNO_3$ were added to different samples together with 100 μg of PNIPAm. When 23% of organic solvent were added into the systems, samples were quenched and examined by TEM, protrusions were growing out from polymersomes. The increase of salt decreased the inner volume of polymersomes, the different at the. Scale bar 500 nm.

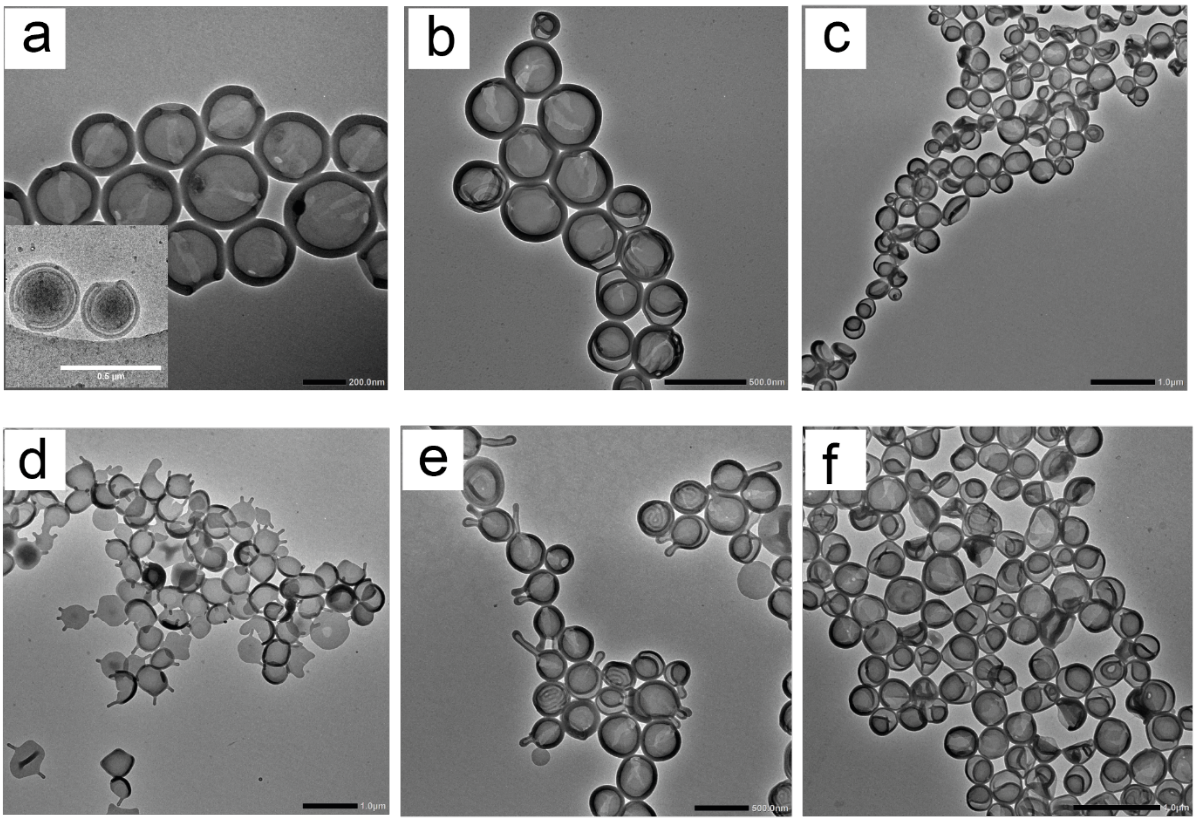

Supplementary Figure 4. Shape transformation of polymersomes with sucrose added to obtain the extra volume reduction. 120 mM sucrose were added to polymersomes to provide extra osmotic pressure during shape transformation, after 23% (a), 31% (b) and 37.5% (c) of organic solvent were added into the systems, samples were quenched and examined by TEM and Cryo-TEM. 120 mM sucrose was then mixed with 250 μg of PNIPAm and added to polymersome solution, when 23% (d), 31% (e) and 37.5% (f) of organic solvent were added into the system, samples were quenched and examined by TEM. Extra inner volume reduction of polymersomes were obtained after sucrose were added into the system, however its effect on the growth of protrusions seemed different from salt ions. Scale bar 500 nm.

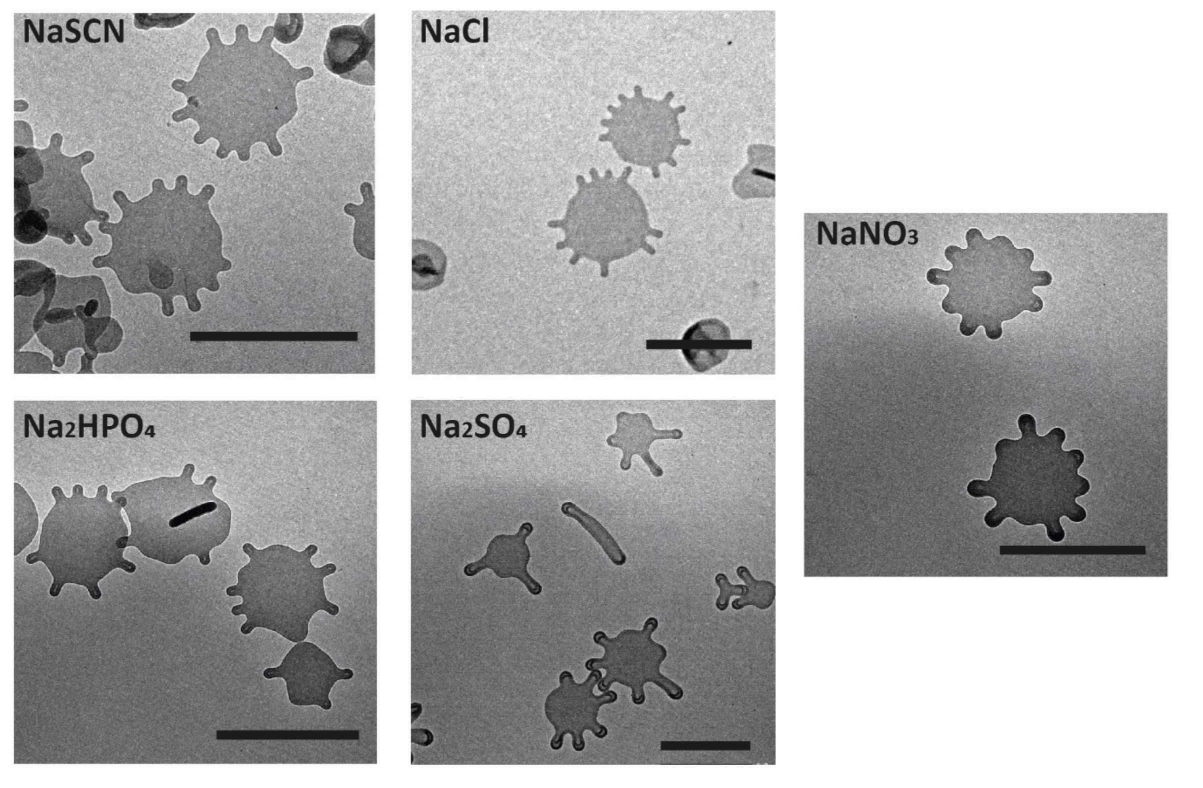

Supplementary Figure 5. Shape transformation of polymersomes with Hofmeister salt. 60 mM NaSCN, NaCl, $NaNO_3$, Na_2HPO_4 and Na_2SO_4 were added to polymersomes together with 250 µg of PNIPAm to explore the salt impact on membrane curvature formation. After the added organic solvent (THF:Dioxane =4:1) reaches 23% of the total sample volume, samples were quenched and examined by TEM. Scale bar 1 µm.

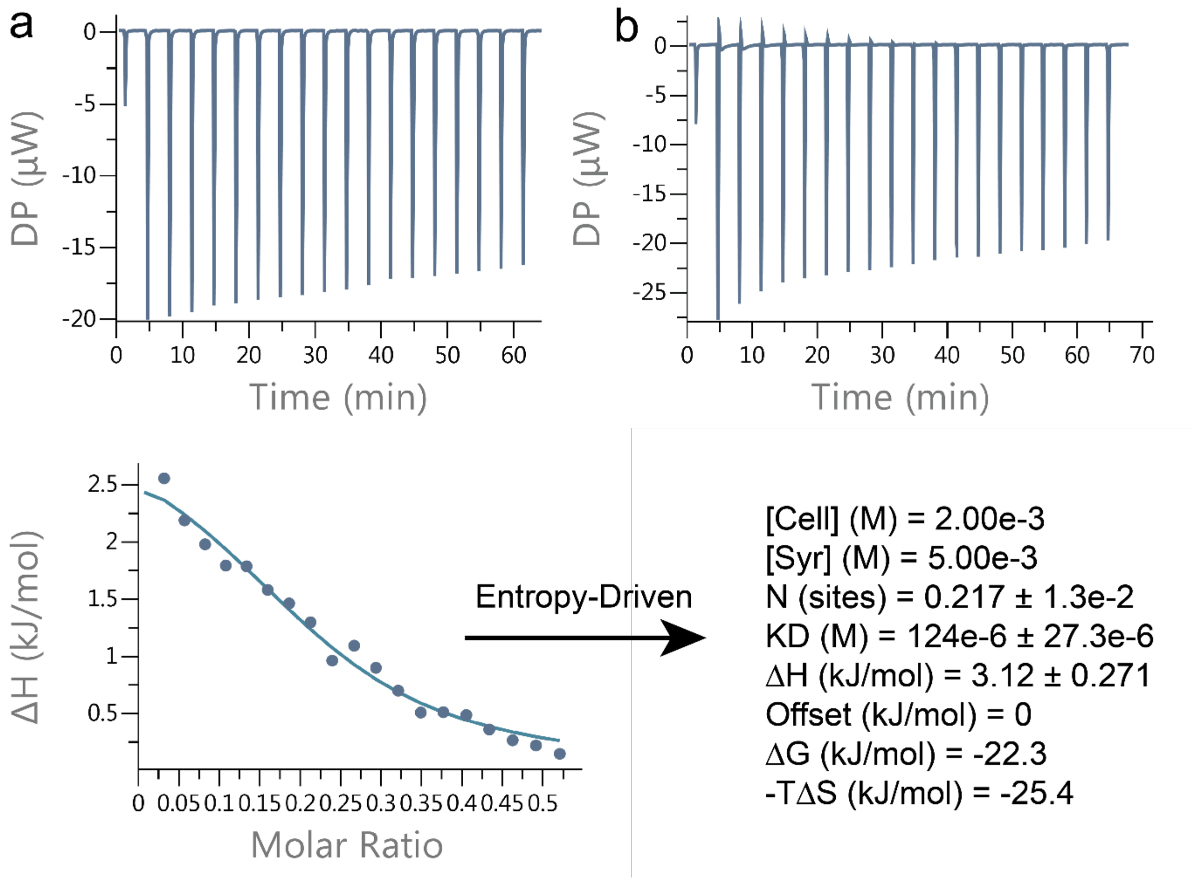

Supplementary Figure 6. ITC measurement PEG-PNIPAm. (a) Raw data and thermograms, sample cell was filled with PEG solution (2 mM), syringe cell was filled with PNIPAm solution (5 mM). (b) Raw data of the dilution of PNIPAm. Error bars indicates the errors of integrated heats,estimated binding affinity (K_d) and binding sites (N).

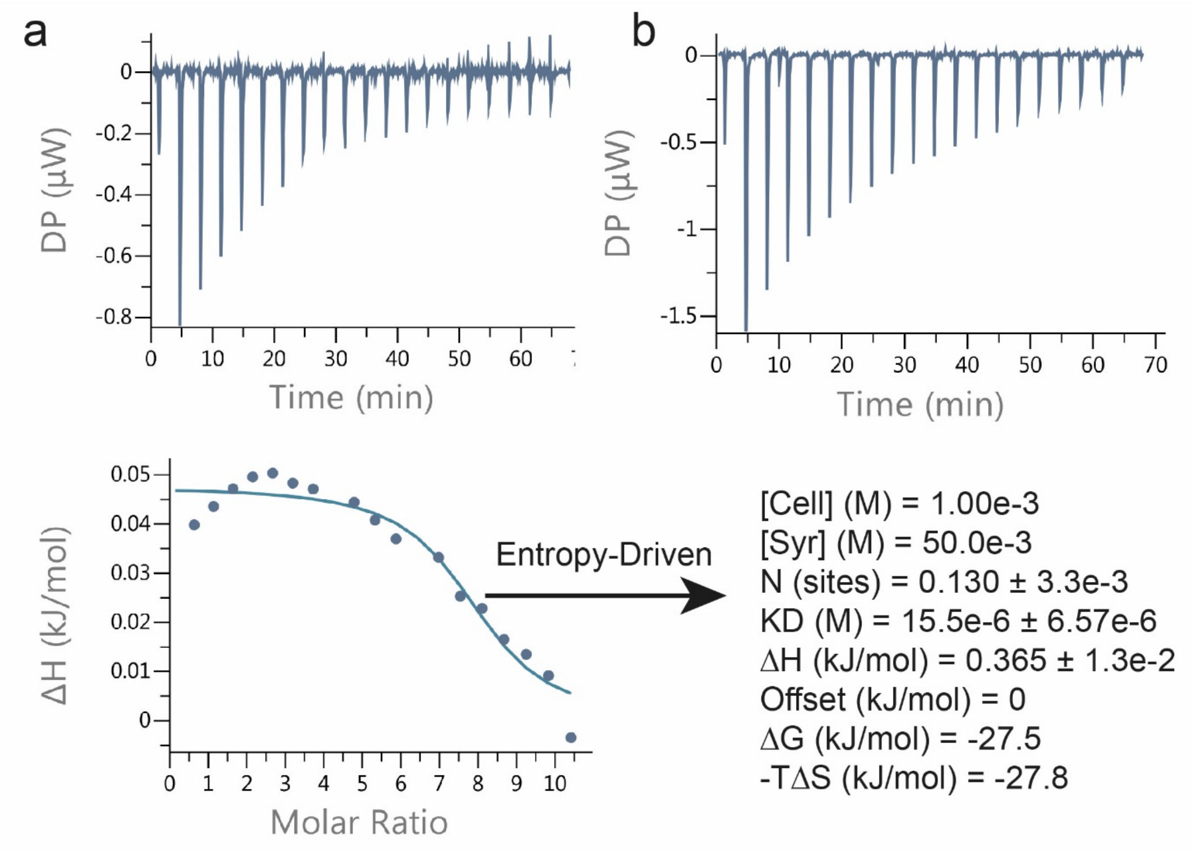

Supplementary Figure 7. ITC measurement PNIPAm-$NaNO_3$. (a) Raw data and thermograms, sample cell was filled with PNIPAm solution (1 mM), syringe cell was filled with $NaNO_3$ solution (50 mM). (b) Raw data of the dilution of $NaNO_3$. Error bars indicates the errors of integrated heats,estimated binding affinity (K_d) and binding sites (N).

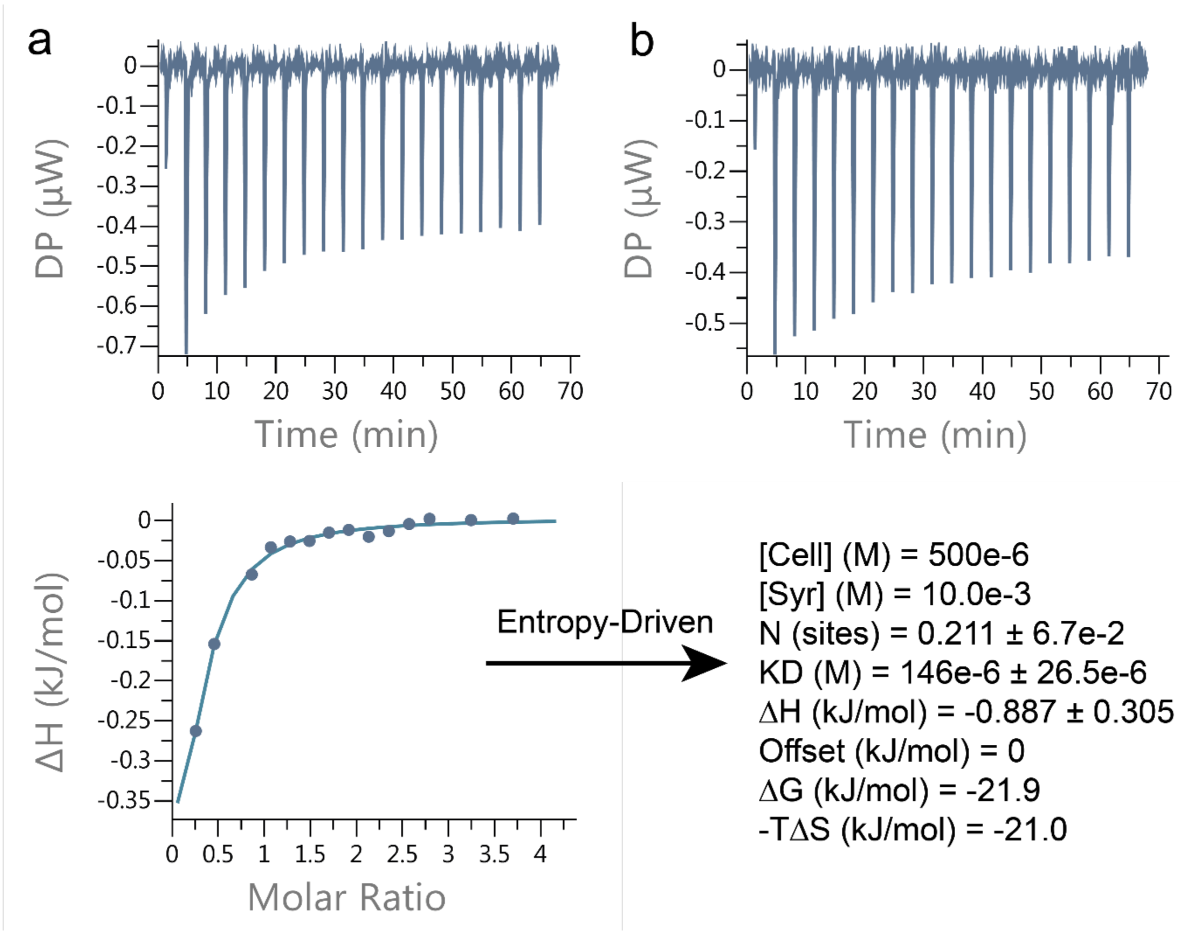

Supplementary Figure 8. ITC measurement Polymersomes-$NaNO_3$. (a) Raw data and thermograms, sample cell was filled with polymersome solution (0.5 mM), syringe cell was filled with $NaNO_3$ solution (10 mM). (b) Raw data of the dilution of $NaNO_3$. Error bars indicates the errors of integrated heats,estimated binding affinity (K_d) and binding sites (N).

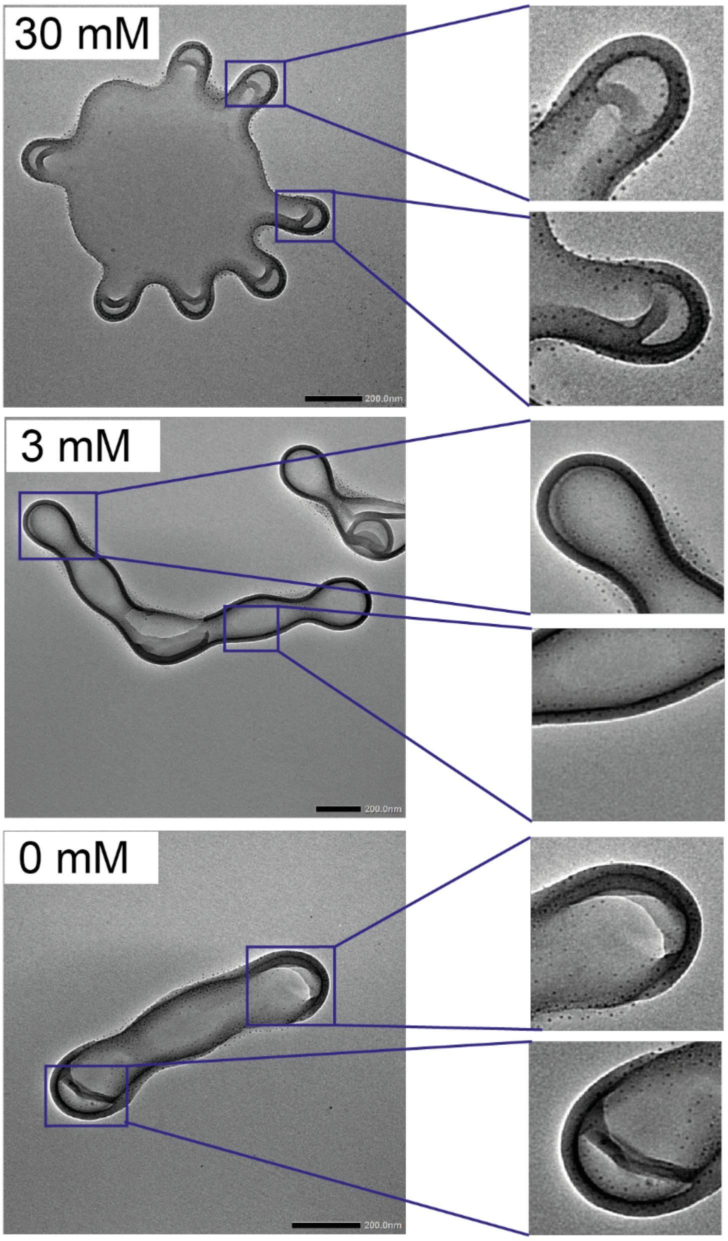

Supplementary Figure 9. Visualization of the PNIPAM distribution in the polymersome membrane after shape transformation with salt using staining method. TEM image of polymersome membrane dyed with copper sulfate ($CuSO_4$), coordination of Cu^{2+} with PNIPAm makes this polymer visible. Scale bar 200 nm.

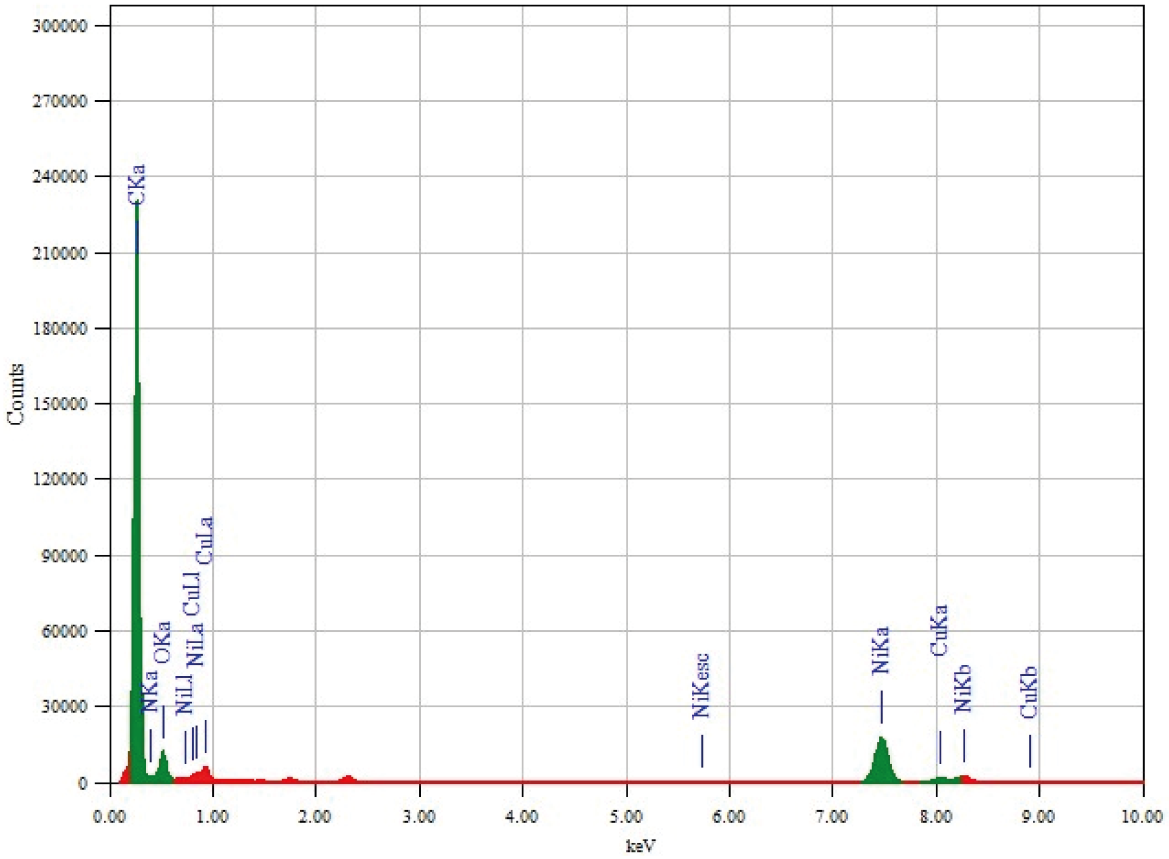

Supplementary Figure 10. EDX spectra of the multi-arm polymersome, C, N, O, Cu, Ni was detected.

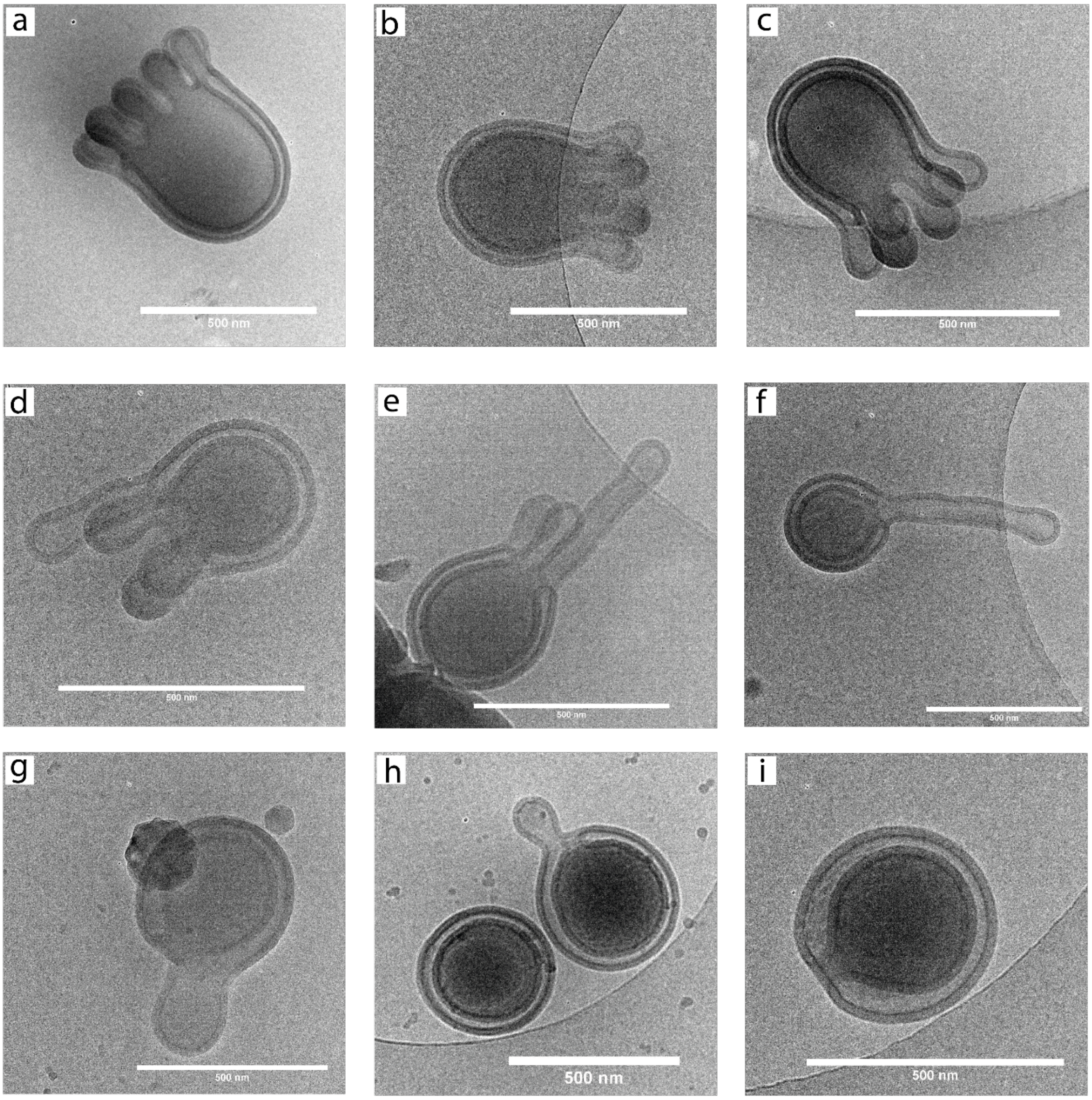

Supplementary Figure 11. Cryo-TEM images of octopus like polymersomes with multiple arms (a—c), 4 arms (d), 3 arms (e), one arm (f - h) and stomatocyte structure control before the shape transformation (i). Scale bar 500 nm.

Supplementary Tables

Supplementary Table 1. Parameterization of different shapes from cryo-TEM images (n=5). For every fitted shape, the reduced volume and area (∆v) are calculated. SD indicates the standard deviation of each value.

**1mM**	**Sample 1**	**Sample 2**	**Sample 3**	**Sample 4**	**Sample 5**	**Average**	**SD**
**ΔV**	0.25	0.33	0.26	0.33	0.27	0.288	0.039
**Δa**	0.36	0.38	0.33	0.49	0.4	0.392	0.061
**3mM**	Sample 1	Sample 2	Sample 3	Sample 4	Sample 5		
**ΔV**	0.28	0.24	0.24	0.21	0.24	0.242	0.025
**Δa**	0.55	0.45	0.45	0.58	0.45	0.496	0.064
**15mM**	Sample 1	Sample 2	Sample 3	Sample 4	Sample 5		
**ΔV**	0.22	0.22	0.18	0.23	0.22	0.214	0.019
**Δa**	0.64	0.34	0.5	0.54	0.34	0.472	0.131
**60mM**	Sample 1	Sample 2	Sample 3	Sample 4	Sample 5		
**DV**	0.19	0.18	0.21	0.15	0.23	0.192	0.030
**DA**	0.42	0.45	0.44	0.48	0.37	0.432	0.041

Supplementary References

1 Rikken, R. S. M. *et al.* Shaping polymersomes into predictable morphologies via out-of-equilibrium self-assembly. *Nature Communications* **7**, 12606 (2016).
